# A Novel Pedicle Screw with Mobile Connection: A Pilot Study

**DOI:** 10.1155/2014/841958

**Published:** 2014-03-03

**Authors:** Yasuaki Tokuhashi, Masashi Oshima, Yasumitsu Ajiro, Hiroshi Uei

**Affiliations:** Department of Orthopaedic Surgery, Nihon University School of Medicine, 30-1 Oyaguchi-kamimachi, Itabashi-ku, Tokyo 173-8610, Japan

## Abstract

To prevent adjacent disc problems after spinal fusion, a pedicle screw with a mobile junction between the head and threaded shaft was newly developed. The threaded shaft of the screw has 10 degrees mobility in all directions, but its structure is to prevent abnormal translation and tilting. This screw was evaluated as follows: (1) endurance test: 10^6^ times rotational stress was applied; (2) biological reactions: novel screws with a mobile head and conventional screws with a fixed head were inserted into the bilateral pedicles of the L3, L4, and L5 in two mini pigs with combination. Eight months after surgery, vertebral units with the screw rod constructs were collected. After CT scan, the soft and bony tissues around the screws were examined grossly and histologically. As a result, none of the screws broke during the endurance test stressing. The mean amount of abrasion wear was 0.0338 g. In the resected mini pig section, though zygapophyseal joints between fixed-head screws showed bony union, the amount of callus in the zygapophyseal joints connected with mobile-head screws was small, and joint space was confirmed by CT. No metalloses were noted around any of the screws. Novel screws were suggested to be highly durable and histologically safe.

## 1. Introduction

Adjacent disc problems after spinal fusion have been considered unavoidable, and the overall annual incidence and predicted 10-year prevalence of further surgery are 2.5% and 22.2%, respectively [1]. As a result, these disorders are never negligible; therefore, to solve these problems, stabilization techniques for spinal instability without rigid intervertebral fusion that preserve intervertebral disc mobility have been developed. Typical examples are artificial intervertebral discs [2, 3] and various dynamic stabilization systems [4–6]. We developed a novel pedicle screw with a mobile metal-on-metal structure at the junction between the head and thread shaft of the screw, which preserved and controlled intervertebral mobility in any direction, and dimples on the contact surface to contain abrasion wear. In this study, the durability of this novel screw and biological reactions to it was evaluated.

## 2. Materials and Methods

### 2.1. Novel Pedicle Screw

**Figure 1:**
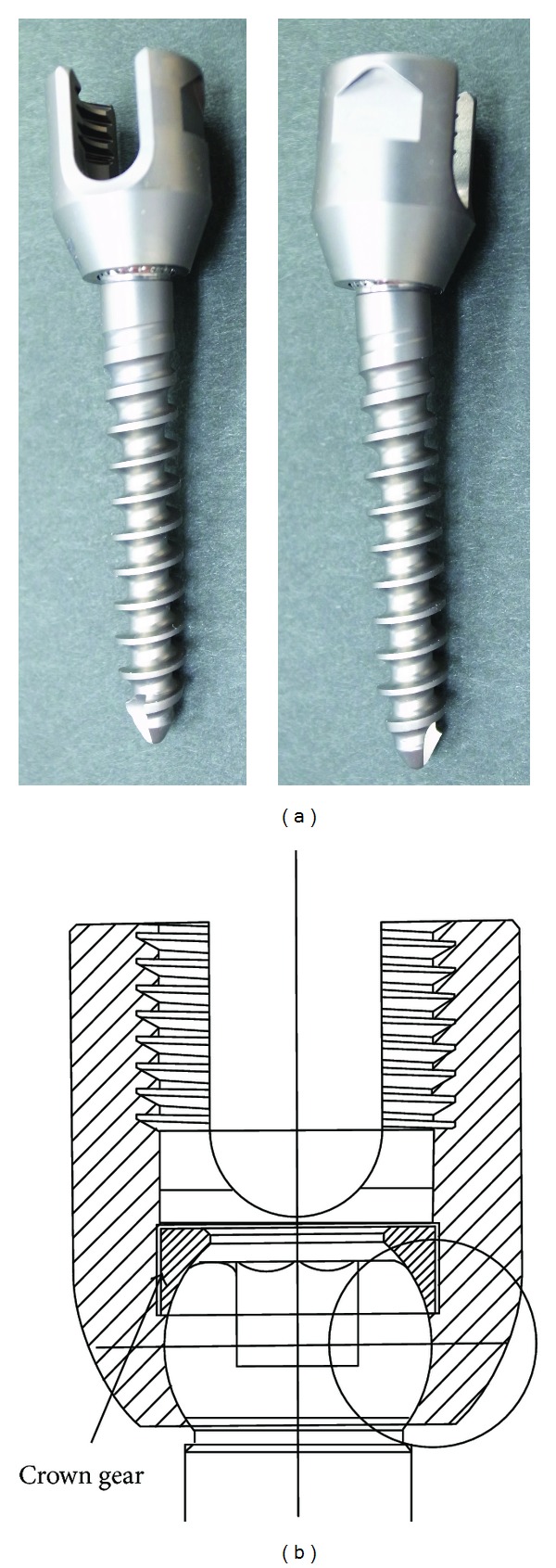
Novel pedicle screw with mobile connection. Junction between the screw head and threaded shaft has a crown gear mechanism and 10° mobility in all directions.

The new pedicle screw is manufactured, which is made of titanium alloy and has a mobile polyaxial structure at the junction between the head and threaded shaft (Figure 1). Because of this mobile structure, the junction of the screw is mobile even after fixation of the rod and head of the screw. The threaded shaft of the screw has 10 degrees mobility in all directions, but its structure is to prevent abnormal movement of vertebral translation and tilting beyond 10 degrees.

**Figure 2:**
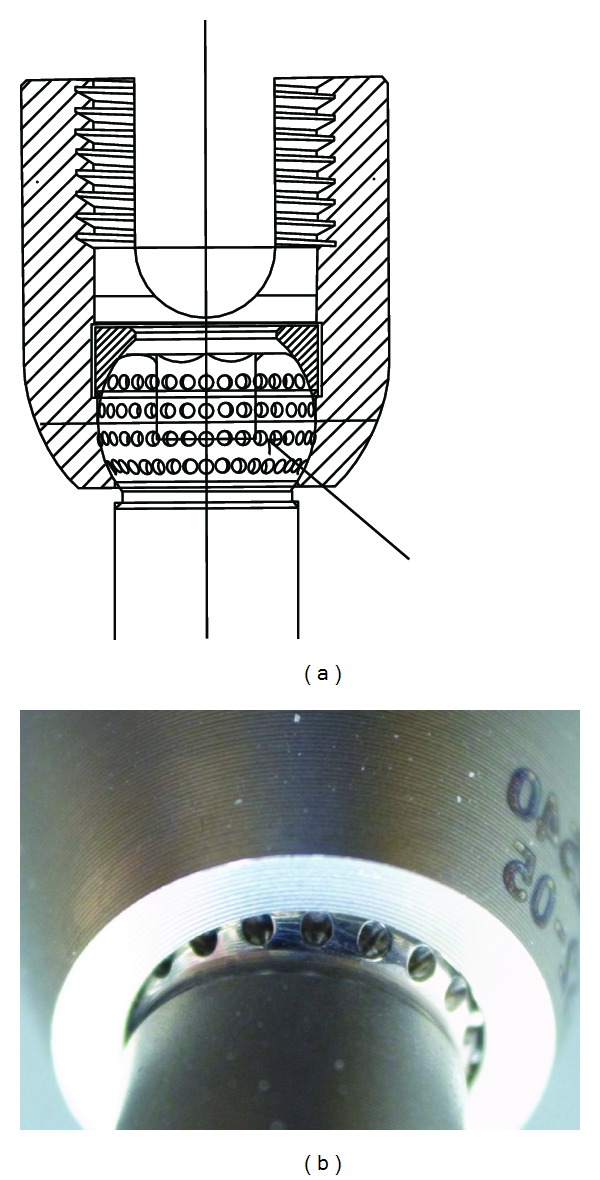
Contact surface between screw head and threaded shaft in novel pedicle screw. Dimples on the contact surface contain the abrasion wear generated after implantation.

Dimples were made on the contact surface of the thread shaft at the junction of the screw to contain the abrasion wear generated after implantation (Figures 1 and 2). The threaded shaft of the screw is 3 conical, with a maximum diameter of 6 mm and 40 mm long, similar to conventional screws.

### 2.2. Durability Testing of the Screw

The mobile head of the novel pedicle screw was fixed in a servo-type fatigue tester from Shimadzu Corporation (Kyoto, Japan), and rotational stress was applied with a motor at an angle of ±5° relative to the threaded shaft, displacement of ±2.2 mm, and frequency of 5 Hz. The stress was applied 10^6^ times, and the room temperature was adjusted to 25°C. Three screws were similarly tested, whether breakage occurred or not, and the amounts of abrasion powder were evaluated. The amount of abrasion wear was calculated by weighing the screw before and after the fatigue test. After durability testing, the screws were washed with acetone and weighed on an electronic balance AW320 (Simadzu). Three screws with no dimples on the contact surface were similarly tested.

### 2.3. Evaluation of Biological Reactions to the Implanted Screws

**Figure 3:**
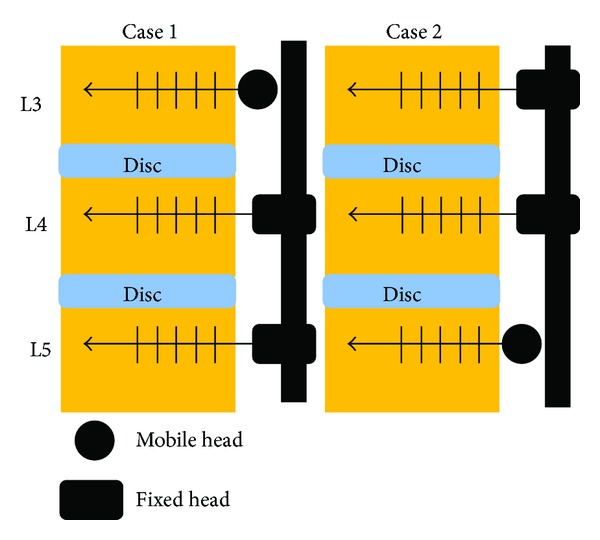
Combination of mobile and fixed-head pedicle screw fixation.

Two 6-month-old domestic minipigs weighing 100 kg were anesthetized and screws were inserted into the bilateral pedicles at L3, L4, and L5 under fluoroscopic guidance. In one minipig, novel screws with a mobile head were inserted at L3, and conventional screws with a fixed head (Globalys; KISCO, Kobe Japan) were inserted at L4 and L5. In the other minipig, conventional screws with a fixed head were inserted at L3 and L4, and novel screws with a mobile head were inserted at L5 (Figure 3). The inserted parts of all screws were conical with a maximum diameter of 6 mm and 40 mm long. After the preparation of the models, the minipigs were cared for over 8 months at a facility specializing in the care of domestic pigs. They were sacrificed 8 months after surgery. After sacrifice, the serum titanium concentration was measured 3 times each using an ICP atomic emission spectrometer, and the values were compared with those before surgery. Five serial vertebral bodies including the intervertebral discs (including the cranial and caudal end plates) and the screws with rods were resected with the surrounding soft tissues as a unit. Each resected unit was scanned by CT. Resin blocks were prepared without removing the screws; the resin block of the vertebral body to the right of each screw was sectioned with a diamond saw, ground, mounted on a glass plate, and examined microscopically. The soft and bony tissues around the screw were examined grossly and histologically.

## 3. Results

### 3.1. Durability Testing

None of the screws tested broke in the predetermined number of trials. The mean amount of abrasion wear, determined by weighing before and after the fatigue test, was 0.0338 g (Table 1(a)), which was about 10% lower than the amount of abrasion wear from the screws with no dimples (Table 1(b)).

### 3.2. Biological Reactions of the Screw Recipient Sites

**Figure 4:**
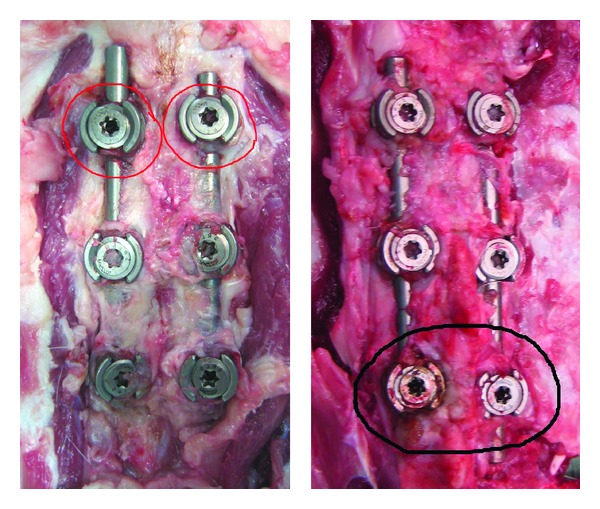
Macroscopic findings. Removed vertebral column unit: circles indicate mobile-head pedicle screws.

**Figure 5:**
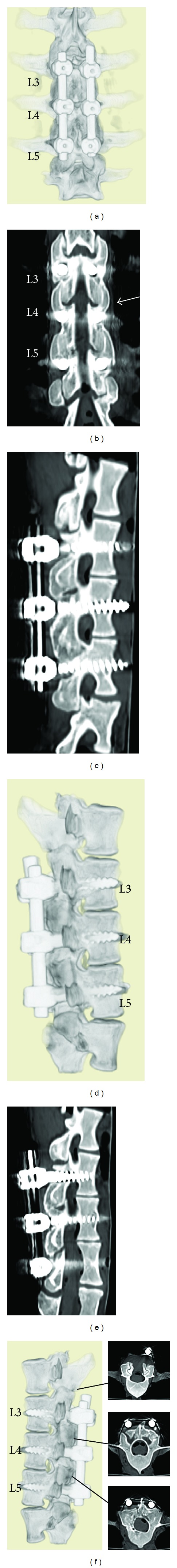
CT findings of removed vertebral column unit. (a) 3D CT: coronal view. (b) Coronal section: bilateral L3/L4 zygapophyseal joints were not fused. (c) Right sagittal section: L4/L5 zygapophyseal joint was not seen, but L3/L4 zygapophyseal joint was clear. (d) 3D CT: sagittal view. (e) Left sagittal section: L4/L5 zygapophyseal joint was obscure, but L3/L4 zygapophyseal joint was clear. (f) Axial section: though bilateral, L4/L5 bilateral zygapophyseal joints were obscure and bilateral L3/L4 zygapophyseal joints were clear.

**Figure 6:**
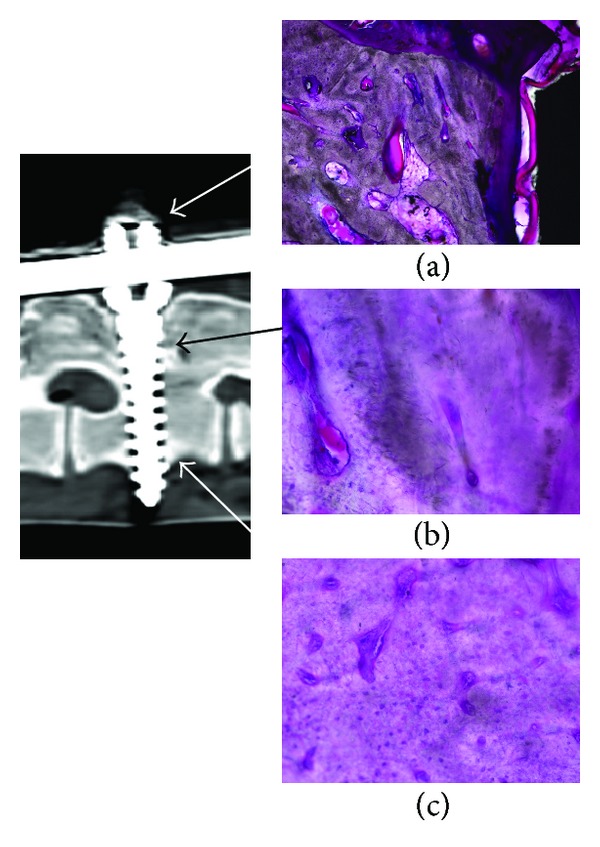
Microscopic findings of tissue around a novel pedicle screw. (a) Bony and granulation tissue near the screw head, (b) bony tissue near junction between screw head and shaft, and (c) bony tissue near screw tip.

No gross metalloses were noted around any of the screws (Figure 4). Bony union was grossly observed between the pedicles of the vertebrae connected with fixed-head screws and was also confirmed by CT scanning (Figure 5). In the spaces between the zygapophyseal joints connected with mobile-head screws, the gross amount of callus was smaller than in those between the vertebrae connected with conventional fixed-head screws, and joint spaces were confirmed by CT scanning (Figure 5). After removal of screws, all the mobile junctions retained its 10 degrees of mobility. The mean serum titanium concentration at sacrifice was 50.8 ± 3.45 and 38.4 ± 1.58 ng/g, respectively (preoperative control value: 15.9 ± 1.80 ng/g). No metalloses were noted by histological examination of the specimens of the vertebral bodies on the right side of the screws prepared from the resin blocks containing the screws (Figure 6).

## 4. Discussion

To solve the problem of adjacent spinal segment disorder after spinal fixation, surgical procedures to treat spinal instability that preserve intervertebral mobility without fusion of vertebral segments have been developed [4–6]. While various dynamic stabilization systems have been proposed, they have been unsatisfactory in the durability and direction of motion control [7–9]. Our novel pedicle screw was developed by adopting a metal-on-metal structure at the junction between the head and threaded shaft. A metal-on-metal structure has already been used in artificial hip joints [10]. Our screws are made of titanium alloy for the convenience of postimplantation MRI examination, but the abrasion wear generated by the contact between metal surfaces has always posed problems [11]; therefore, we made dimples on the contact surface to contain the abrasion wear. Such dimples have already been used widely to improve slide ability between metals by containing the abrasion wear due to contact. In the animal experiment using minipigs, no bony union was observed in the zygapophyseal joints connected by these novel screws, and the mobility of the joints was preserved (Figures 4 and 5). The pedicles of minipigs are relatively large and the usual sized screws can be applied; therefore, minipigs have a potential for examining biological reactions to pedicle screws [12]. However, a minipig is not an upright animal and this one is not appropriate in the viewpoint of vertebral loading characteristic. From a problem of the cost-effectiveness, we could use only two minipigs in this experiment. Therefore, we were going to increase numbers of minipigs when they died early or when they did not get along well. Fortunately, they lived safely for eight months. As a result, no metalloses, which had been a concern, were noted grossly or histologically. These results suggest that this novel mobile-head screw shows high-level durability and histological safety; however, as the serum titanium level increased to 2-3 times the preimplantation control level, sufficient follow-up is considered necessary. It is known that the concentrations in the metal serum increase by size and the number of internal implants [13]. Also, though the long-term course is unknown, it is reported that the concentrations in the metal serum do not increase gradually at least with age [14]. The significance of serum metal concentration also remains unclear, so further studies are also necessary. Whether the mobility-preserving effect of the screw maintained over a long period is unclear or not, this novel pedicle screw may be useful for mobile stabilization. It is thought that longer periods of evaluation may be desirable, considering the development of screw loosening.

## Figures and Tables

**Table 1a:** (a)

Time	Pretest (g)	Posttest (g)	Differences (g)
First	9.7427	9.7133	0.0294
Second	9.6567	9.6189	0.0378
Third	9.6340	9.5999	0.0341

Average			0.0338

**Table 1b:** (b)

Time	Pretest (g)	Posttest (g)	Differences (g)
First	9.7375	9.6967	0.0408
Second	9.7518	9.7185	0.0333
Third	9.7214	9.6834	0.0380

Average			0.0374
